# Prognostic power of conventional echocardiography in individuals without history of cardiovascular diseases: A systematic review and meta-analysis

**DOI:** 10.6061/clinics/2021/e2754

**Published:** 2021-06-23

**Authors:** Luciana Pereira Fernandes, Ana Terra Fonseca Barreto, Mansueto Gomes Neto, Edmundo José Nassri Câmara, André Rodrigues Durães, Leonardo Roever, Roque Aras-Júnior

**Affiliations:** I Programa de Pos Graduacao em Medicina e Saude, Universidade Federal da Bahia, Salvador, BA, BR; II Departamento de Ecocardiografia, Hospital Universitario Professor Edgar Santos (HUPES), Universidade Federal da Bahia, Salvador, BA, BR; III Grupo de Pesquisa em Fisioterapia, Universidade Federal da Bahia, Salvador, BA, BR; IV Departamento de Medicina, Universidade Federal da Bahia, Salvador, BA, BR.; V Departamento de Pesquisa Clinica, Universidade Federal de Uberlandia, Uberlandia, MG, BR

**Keywords:** Echocardiography, Risk, Cardiovascular Disease, Cerebrovascular Accident, Mortality

## Abstract

Echocardiographic abnormalities are associated with a higher incidence of adverse cardiovascular outcomes. This systematic review and meta-analysis aimed to evaluate whether echocardiographic abnormalities are predictors of cardiovascular events in individuals without previous cardiovascular diseases. The PubMed, Scopus, and SciELO databases were searched for longitudinal studies investigating the association between echocardiographic abnormalities and cardiovascular events among individuals without known cardiovascular diseases. Two independent reviewers analyzed data on the number of participants, age and sex, echocardiographic alterations, follow-up time, and cardiovascular outcomes. The meta-analysis estimated the risk ratio (RR) and 95% confidence interval (CI). Heterogeneity was assessed using I^2^ test. Twenty-two longitudinal studies met the eligibility criteria, comprising a total of 55,603 patients. Left ventricular hypertrophy (LVH) was associated with non-fatal cardiovascular events (RR 2.16; 95% CI 1.22-3.84), death from cardiovascular disease (RR 2.58; 95% CI 1.83- 3.64), and all-cause mortality (RR 2.02; 95% CI 1.34-3.04). Left ventricular diastolic dysfunction (LVDD) and left atrial dilation (LA) were associated with fatal and non-fatal cardiovascular events (RR 2.01; 95% CI 1.32-3.07) and (RR 1.78; 95% CI 1.16-2.73), respectively. Aortic root dilation was associated with non-fatal cardiovascular events (RR 1.25; 95% CI 1.09-1.43). In conclusion, LVH, LVDD, dilations of the LA, and of the aortic root were associated with an increased risk of adverse events in individuals without previous cardiovascular diseases. This study suggests that simple data obtained on conventional echocardiography can be an important predictor of cardiovascular outcomes in a low-risk population.

## INTRODUCTION

Cardiovascular diseases are the subject of extensive research because of their significant impact on morbidity and mortality worldwide (1). Echocardiography plays an important role in the initial investigation of cardiovascular risk and accounts for a high volume of cardiac imaging tests performed (2). Easy access and non-invasiveness have facilitated its widespread application, and has also increased the detection and diagnosis of several abnormalities in individuals without cardiovascular disease (3).

In this context, studies have reported different results regarding the prognostic impact of echocardiographic abnormalities in asymptomatic individuals (4,5). To the best of our knowledge, no previous systematic review has analyzed echocardiographic abnormalities as predictors of cardiovascular risk. Accordingly, this systematic review and meta-analysis aimed to analyze published studies that investigated whether echocardiographic abnormalities are predictors of adverse cardiovascular outcomes in patients without previous cardiovascular diseases (*e.g.*, myocardial infarction [MI], heart failure [HF], stroke, and atrial fibrillation [AF]).

## METHODS

This systematic review was performed in accordance with the Preferred Reporting Items for Systematic Reviews and Meta-Analyses (PRISMA) guidelines (6). The review protocol was registered in the PROSPERO database (CRD42018117704). Given the retrospective nature of the study (*i.e.*, review and meta-analysis) and the use of anonymized data, the requirement for informed consent was waived.

### Eligibility criteria

Longitudinal studies that addressed the association between echocardiographic abnormalities and adverse cardiovascular outcomes, including fatal and non-fatal MI, fatal and non-fatal stroke, HF, AF, and all-cause mortality in adults >18 years of age were included. Studies using other diagnostic imaging methods that analyzed other outcomes or whose participants had already experienced one of the outcomes were excluded.

Eligible studies were identified by searching the PubMed, Scopus, and Scientific Electronic Library Online (SciELO) databases up to December 2019 without language or publication status restrictions. Manual searches of relevant studies were also performed using *Google Scholar*. A standard protocol was used for the literature search and, whenever possible, using controlled vocabulary (*i.e.*, MeSH term for PubMed). In the search strategy, three groups of keywords and their synonyms were used: study design, participants, and exposure. The optimally sensitive search strategy was used to identify studies in MEDLINE and PubMed. The full search strategy is presented in Table S1.

### Data extraction

Two members of the study team (LPF and ATFB) independently evaluated a list of tittles and abstracts from each data source to identify potentially eligible studies for the systematic review. If at least one of the members considered a reference eligible, a full-text article was obtained for complete assessment.

Data regarding the following variables were extracted from each study: first author, year and country of publication, number of participants, age and sex, echocardiographic parameters analyzed, follow-up, and outcomes evaluated (Table 1). The extracted data were independently verified (*i.e.*, double verification) by two members of the study team (LPF and ATFB). Disagreements were resolved by consensus or discussion with a third investigator (MGN).

### Study quality

The Newcastle-Ottawa Quality Assessment Scale (7) for cohort studies was used to assess the quality of the included studies. The scale consisted of three categories: 1. Selection (sample representativeness, selection of the unexposed cohort, determination of exposure, and absence of selection bias); 2. Comparison between groups; 3. Outcome (analysis of outcomes, follow-up time, and adequacy of follow-up time). The total quality score was reported as the average score of the two researchers.

### Statistical analysis

Risk ratio (RR) was calculated from the number of events and participants in each group (left ventricular diastolic dysfunction [LVDD] *versus* *[vs.]* control, left ventricular hypertrophy [LVH] *vs*. control, left atrial [LA] enlargement *vs*. control, and aortic root dilation *vs*. control) and used to compare dichotomous variables. Cardiovascular events (fatal and non-fatal), cardiovascular mortality, and all-cause mortality were analyzed. Pooled RR was calculated.

All *p*-values were two-tailed, with a statistical significance of 0.05, and confidence intervals (CIs) were calculated at the 95% level. The RR and 95% CIs were calculated. The heterogeneity of the treatment effect in the meta-analysis was examined using the I^2^ statistic. The I^2^ values >40% were considered to be indicative of high heterogeneity and, in this case, a random-effects model was chosen. Meta-analysis was conducted using Review Manager (version 5.3; Copenhagen: The Nordic Cochrane Center, The Cochrane Collaboration) (8). We only included the studies that presented the same type of exposure in the meta-analysis and analyzed the same outcomes.

## RESULTS

### Description of selected studies

**Figure 1:**
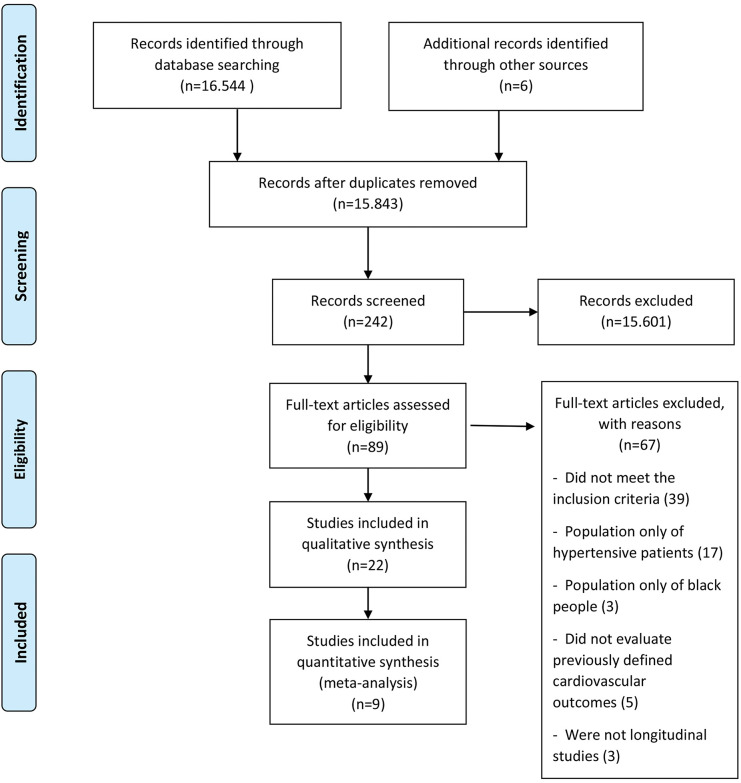
PRISMA flow diagram showing study identification, selection, eligibility, and inclusion.

The initial search retrieved 16,544 abstracts, of which 89 studies were considered potentially relevant and were included in the detailed analysis. Ultimately, 22 studies (9-30) met the eligibility criteria, and nine were included in the meta-analysis (13-16,18,20,26,27,29). The PRISMA flow diagram of the studies in this review is presented in Figure 1. The results of the assessment of the Newcastle-Ottawa Quality Assessment Scale are presented individually in Table S2.

### Study characteristics

The studies were conducted in seven countries between 1990 and 2019, including a total of 55,603 patients in the systematic review and 24,639 patients in the meta-analysis. There was a predominance of females (55.6%), with an average age of 55.4 years. Most studies included adults >40 years of age, six elderly individuals, three healthy young individuals, and two evaluated Native Americans. North American population studies (14 studies) predominated, followed by six European and two Chinese studies. The mean follow-up period was 10.4 years. All studies, except for two (17,24), were prospective cohorts, and all were adjusted for possible confounding variables (sociodemographic, clinical, or echocardiographic) using multivariate analysis. The characteristics of the included studies are summarized in Table 1.

### The effects of echocardiographic abnormalities on clinical results

Four studies (9-11,14) that analyzed the association between left ventricular (LV) geometry and the incidence of adverse cardiovascular outcomes reported that individuals with concentric or eccentric hypertrophy had higher cardiovascular risk.

Regarding the association between LV mass and cardiovascular outcomes, Armstrong et al. (12) found that adding LV mass to the Framingham score modestly increased discrimination. Lai et al. (13) found a significant association among Chinese individuals. Levi et al. (15) observed an association between an increase in LV mass and a higher risk of adverse cardiovascular outcomes in the Framingham cohort.

**Figure 2.1:**
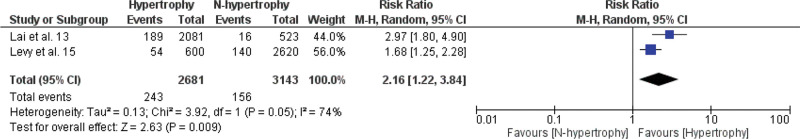
The Risk Ratio and 95% confidence interval (CI) in the cardiovascular events (non-fatal), for Left Ventricular Hypertrophy group *versus* the Control group.

**Figure 2.2:**
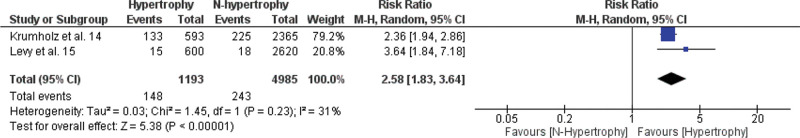
The Risk Ratio and 95% confidence interval (CI) in the cardiovascular mortality, for Left Ventricular Hypertrophy group *versus* the Control group.

**Figure 2.3:**
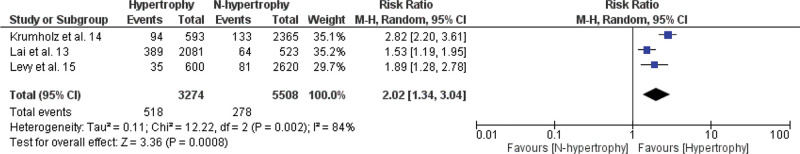
The Risk Ratio and 95% confidence interval (CI) in the all-cause mortality, for Left Ventricular Hypertrophy group *versus* the Control group.

A meta-analysis of the association between LVH and non-fatal cardiovascular events, fatal cardiovascular events, and all-cause mortality is shown in Figures 2.1, 2.2, and 2.3. The RR of the associations of LVH with non-fatal cardiovascular events, fatal cardiovascular events and with mortality from all causes were: 2.16 (95% CI 1.22-3.84), 2.58 (95% CI 1.83-3.64), and 2.02 (95% CI 1.34-3.04), respectively, indicating a significantly increased risk for these events in the LVH group *versus* the non-LVH group (*p*<0.01).

**Figure 3.1:**
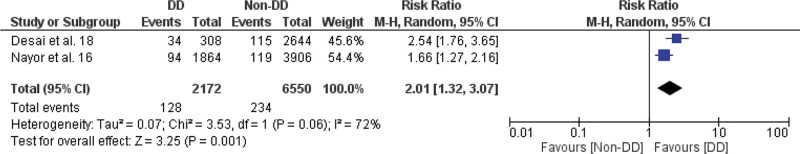
The Risk Ratio and 95% confidence interval (CI) in the cardiovascular events (fatal and non-fatal), for Left Ventricular Diastolic Dysfunction group *versus* the Control group.

**Figure 3.2:**
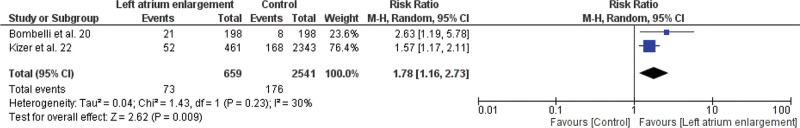
The Risk Ratio and 95% confidence interval (CI) in the cardiovascular events (fatal and non-fatal), for Left Atrial enlargement group *versus* the Control group.

**Figure 3.3:**
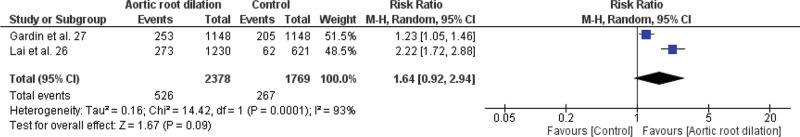
The Risk Ratio and 95% confidence interval (CI) in the all-cause mortality, for aortic root dilation group *versus* the Control group.

**Figure 3.4:**
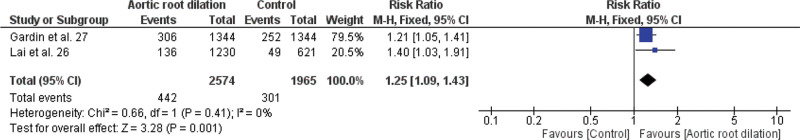
The Risk Ratio and 95% confidence interval (CI) in the cardiovascular events (non-fatal), for aortic root dilation group *versus* the Control group.

Meta-analysis of the association between LVDD and cardiovascular events (fatal and non-fatal), LA enlargement and cardiovascular events (fatal and non-fatal), aortic root dilation and all-cause mortality, aortic root dilation, and non-fatal cardiovascular events are shown in Figures 3.1, 3.2, 3.3, and 3.4, respectively.

Among the studies that evaluated LVDD (16-19), Nayor et al. (16) assessed the impact of age- and sex-specific criteria on the diagnosis of LVDD and found that, based on these criteria, LVDD assessment was less age-dependent and more associated with incidental cardiovascular disease. Aljaroudi et al. (17) evaluated the incremental prognostic value of LVDD in the Framingham risk score and observed that, even after adjusting for age, sex, and race, LVDD remained an independent predictor of death from all causes. Desai et al. (18) evaluated the prevalence and prognosis of LVDD in young adults and found that LVDD was associated with high cardiovascular morbidity and mortality. Finally, in a cohort of the Rotterdam Study, Kardys et al. (19) found that asymptomatic individuals with LVDD had a higher risk of death from all causes.

In this study, we found a significant increase in the risk of fatal and non-fatal cardiovascular events in the LVDD group compared to the control group, with an RR of 2.01 (95% CI 1.32-3.07), as shown in Figure 3.1.

Five studies assessed the association between LA dilation and the risk of cardiovascular events (20-24). Bombelli et al. (20) found that LA dilation was an independent factor for the incidence of cardiovascular events in the general population. In contrast, Armstrong et al. (21) did not observe any increase in cardiovascular risk prediction when LA dimensions were added to the Framingham risk score. Kizer et al. (22) found that LA dilation was an independent predictor of first cardiovascular events in a population of middle-aged and elderly adults. Laukkanen et al. (23) did not find a statistically significant association between LA dilation and cardiovascular mortality after adjusting for LV mass in a cohort of middle-aged men. Finally, Tsang et al. (24) found that LA dilation was a robust predictor of first cardiovascular events in the elderly population.

As seen in Figure 3.2, we observed in this study a significantly increased risk of cardiovascular events in the group with increased LA compared to the control group with RR of 1.78 (95% CI 1.16-2.73).

Three studies estimated the association between aortic root dilation and cardiovascular risk (25-27). Cuspidi et al. (25) found a significant association between height-indexed aortic root dilation and cardiovascular risk in middle-aged individuals. Lai et al. (26) observed an increase in the incidence of cardiovascular events in a Chinese population aged <65 years with aortic root dilation. Finally, Gardin et al. (27) observed that elderly individuals with aortic root dilation demonstrated an increased risk of cardiovascular events, except for acute MI.

In this study, we found no association between dilation of the aortic root and mortality from all causes, with RR=1.64; 95% CI 0.92-2.94 (Figure 3.3), but we found in relation to non-fatal cardiovascular events, with RR=1.25; 95% CI 1.09-1.43 (Figure 3.4).

Three studies analyzed the association between aortic valve (AV) sclerosis and mitral annular calcification (MAC) and the risk of cardiovascular events in healthy individuals (28-30). Völzke et al. (28) found that both AV sclerosis and MAC increased the risk of all-cause and cardiovascular mortality. Kizer et al. (29) reported an increased risk of stroke among Native Americans with MAC, but not among those with AV sclerosis. Finally, Gardin et al. (30) concluded that MAC was a predictor of incidental coronary heart disease in an elderly cohort. However, it was not possible to perform meta-analyses in these studies.

## DISCUSSION

Echocardiography is a non-invasive and easy-to-perform test that can detect many changes in cardiac structure and function frequently associated with cardiovascular prognosis in different situations, and it should also be used for this purpose.

To the best of our knowledge, this is the first meta-analysis to specifically investigate the role of some typical parameters of a conventional echocardiogram in a long-term prognosis in individuals without known previous cardiovascular events, including a total of 55,603 patients in the systematic review and 24,639 in the meta-analysis.

LVH, LVDD, LA enlargement, and aortic root dilation were associated with an incremental risk of adverse cardiovascular outcomes in this population, including fatal and non-fatal events, as well as all-cause mortality for LVH.

LVH and geometric patterns have long been recognized as predictors of increased cardiovascular risk. A meta-analysis published in 2001 reported that patients with LVH had twice the risk of cardiovascular events and death, regardless of other risk factors (31). Our findings are consistent with this finding, even in an asymptomatic population.

An important finding of this meta-analysis is the significant association of any grade of diastolic dysfunction with fatal and non-fatal cardiovascular events in individuals without known previous cardiovascular diseases. This reinforces the view that the finding of LVDD should not be directed towards the diagnosis of HF, but should also be valued as an early marker of cardiovascular prognosis.

LVDD is typically seen in patients with hypertension but can also occur in a variety of other clinical disorders and has a particularly high prevalence in the elderly population (32). The association between LVDD and fatal and non-fatal cardiovascular events that we found was also seen by Seko et al. (33), who, found a statistically significant association between LVDD and all-cause mortality. Other community studies involving the general population also demonstrated a significant predictive value of LVDD for cardiovascular events and mortality (34,35).

LA enlargement is a highly valued finding in echocardiography, which provides supportive evidence of structural alteration(s) of the heart. It is a parameter of LVDD and has been considered a predictor of cardiovascular events, HF, arrhythmias (atrial fibrillation), and mortality (20-24). Froehlich et al. (36) found that LA enlargement is associated with cardiovascular outcomes in patients with and mainly without atrial fibrillation. According to the authors, LA myopathy is the main cause of these results. This study reinforces our finding that LA enlargement is significantly associated with cardiovascular events in individuals without previous cardiovascular diseases.

Concerning aortic root dilation, our results are consistent with those of the Framingham Heart Study cohort (37) which found that aortic root remodeling was associated with the risk of HF with reduced ejection fraction.

An important contribution of our review stems from the fact that echocardiographic abnormalities were diagnosed using simple and widely available methods (Table S3). Thus, these parameters can be used to assess the risk of cardiovascular outcomes in most echocardiography services without requiring more sophisticated techniques, benefiting a larger number of patients.

The findings of this study may have important implications for clinical practice because it increases awareness that individuals with echocardiographic abnormalities, even those who are asymptomatic and without previous diseases, should be monitored more carefully.

Two questions are presented here for discussion. LVH, LVDD, LA enlargement and also aortic dilation often occur together, and it is not possible to separate in a meta-analysis study like ours, the weight and independence of each of these parameters, as well as the association of some of them, on future cardiovascular disease events. Studies are needed to create a risk score associated with these factors. Another problem is that many of the studies that make up this meta-analysis used the m-mode to estimate the mass, measure the LA and the diameter of the aortic root. Likewise, for the diastolic function, some studies did not use tissue Doppler and other criteria according to the new guidelines. Therefore, it is possible that using more recent validated methods, different predictive values, and perhaps more significant RRs, can be found.

Currently, the concept of multimodal imaging has become increasingly reinforced (38). Two important biomarkers/predictors of cardiovascular risk are the coronary artery calcium score and the ratio of carotid artery intima-media thickness/atherosclerotic plaques (39,40). It is necessary to investigate whether some of the echocardiographic parameters described here using appropriate methods are additive for these biomarkers/predictors.

Our study had some limitations. First, the reviewed articles used different echocardiographic approaches, thus increasing heterogeneity in the meta-analysis. Second, only 9 of 22 studies could be selected for meta-analysis, as there were few studies addressing the same exposure or with the same outcomes that could be grouped in the same meta-analysis. In this sense, further research is warranted to assess the contribution of echocardiography in monitoring asymptomatic patients without known previous cardiovascular events.

## CONCLUSIONS

This systematic review and meta-analysis revealed that LVH, LVDD, LA enlargement, and aortic root dilation were associated with an increased risk of adverse cardiovascular outcomes in individuals without known cardiovascular diseases. These findings are important because they confirm the clinical value of monitoring patients with echocardiographic abnormalities to prevent major cardiovascular events, using simple data obtained on conventional echocardiography, even if further studies using more homogeneous methods, populations, and outcomes are still needed.

## AUTHOR CONTRIBUTIONS

Bin KJ, Higa N, Silva J, Quagliano DA, and Ono SK were responsible for the study conception/design, data acquisition, analysis and interpretation, manuscript drafting, review, and final approval. Hangai RK, Cobello-Junior V, Pereira AJR, Carneiro D’Albuquerque LA, Carrilho FJ, and Wen CL were responsible for the data analysis and interpretation, manuscript drafting, review, and final approval.

## Figures and Tables

**Table 1:** Characteristics of the included studies.

Study	Patients (N analyzed, age, gender)	Echocardiographic parameter	Follow-up (years)	Outcomes (N)
Lind et al. (9)	N=1016, 70 years, 50% female	LVH	10	CVD (MI, stroke, HF)
Desai et al. (10)	N=2577, 72.5 years, 64% female	LVH	10	CVD (HF, MI)
Lieb et al. (11)	N=2604, 51 years, 59% female	LVH	12	CVD (MI, HF), CV death
Armstrong et al. (12)	N=3980, 30 years, 54% female	LVH	20	CVD (HF, MI, stroke), CV death
Lai et al. (13)	N=2604, 54.2 years, 54% female	LVH	14.4*	CVD (MI and stroke) all-cause mortality
Krumholz et al. (14)	N=3216, 55.8 years, 56% female	LVH	7.7	CVD, CV death and all-cause mortality
Levy et al. (15)	N=3220, 55.7 years, 56% female	LVH	4	CVD CV death and all-cause mortality
Nayor et al. (16)	N=2355, 44 years, 66% female	LVDD	7.9	CVD (MI, HF, stroke) CV death
AlJaroudi et al. (17)	N=1039, 47.9 years, 73% female	LVDD	7.3	All-cause mortality
Desai et al. (18)	N=2952, 25.2 years, 54% female	LVDD	20	CVD (MI, HF, Stroke) all-cause mortality
Kardys et al. (19)	N=4425, 71.4 years, 61% female	LVDD	3	All-cause mortality
Bombelli et al. (20)	N=1785, 50.6 years, 49.1% female	LA dilation	12.3	CVD CV death and all-cause mortality
Armstrong et al. (21)	N=4082, 30 years, 54% female	LA dilation	20	CVD and CV death
Kizer et al. (22)	N=2804, 59.2 years, 64.4% female	LA dilation	7	CVD and CV death
Laukkanen et al. (23)	N=830, 50.5 years, 100% male	LA dilation	13	CV death
Tsang et al. (24)	N=1160, 75 years, 64% female	LA dilation	3.8	CVD (AF, HF, MI, stroke), CV death
Cuspidi et al. (25)	N=1860, 50 years, 49.4% female	Ao root dilation	12.3	CVD (CHD, HF, stroke), CV death
Lai et al. (26)	N=1851, 57.5 years, 56% female	Ao root dilation	11.9*	CVD, CV death and all-cause mortality
Gardin et al. (27)	N=3933, 72.8 years, 57.6% female	Ao root dilation	10.5	CVD (HF, MI, stoke), CV death and all-cause mortality
Völzke et al. (28)	N=2081, 65 years, 51% female	AV sclerosis and MAC	8.6*	CV death All-cause mortality
Kizer et al. (29)	N=2723, 59.2 years, 64.9% female	AV sclerosis and MAC	7*	Stroke
Gardin et al. (30)	N=2506, 73 years, 65% female	MAC	6.5	CVD (CHD, stroke, HF) All- cause mortality

* median; LVH, left ventricle hypertrophy; CVD, cardiovascular disease; MI, myocardial infarction; HF, heart failure; CV, cardiovascular; LVDD, left ventricular diastolic dysfunction; LA, left atrium; Ao, aortic; CHD, coronary heart disease; AV, aortic valve; MAC, mitral annular calcification.

## APPENDIX SUPPLEMENTARY MATERIAL

**Table S1:** The full search strategy MEDLINE/PUBMED. No Language, article type, or publication date restrictions.

1-	((proportional hazard models) OR (hazard model, proportional) OR (hazard models, proportional) OR (model, proportional hazard) OR (models, proportional hazard) OR (proportional hazard model) OR (models, proportional hazards) OR (hazards model, proportional) OR (hazards models, proportional) OR (model, proportional hazards) OR (proportional hazards model) OR (hazards models) OR (hazards model) OR (model, hazards) OR (models, hazards) OR (hazard models) OR (hazard model) OR (model, hazard) OR (models, hazard) OR (cox proportional hazards models) OR (cox models) OR (models, cox) OR (risks) OR (relative risk) OR (relative risks) OR (risk, relative) OR (risks, relative) OR (odds ratios) OR (cross-product ratio) OR (cross-product ratio) OR (cross-product ratios) OR (ratio, cross-product) OR (ratios, cross-product) OR (relative odds) OR (odds, relative) OR (risk ratio) OR (ratio, risk) OR (ratios, risk) OR (risk ratios) OR (incidence) OR (“Incidence”[Mesh]) OR (“Risk”[Mesh]) OR (“Odds Ratio”[Mesh]) OR (“Proportional Hazards Models”[Mesh])).
2-	((“Cohort Studies”[Mesh]) OR (“Longitudinal Studies”[Mesh]) OR (cohort study) OR (studies, cohort) OR (study, cohort) OR (concurrent studies) OR (studies, concurrent) OR (concurrent study) OR (study, concurrent) OR (closed cohort studies) OR (cohort studies, closed) OR (closed cohort study) OR (cohort study, closed) OR (study, closed cohort) OR (studies, closed cohort) OR (analysis, cohort) OR (cohort analysis) OR (analysis, cohort) OR (cohort analyses) OR (historical cohort studies) OR (cohort study, historical) OR (historical cohort study) OR (study, historical cohort) OR (cohort studies, historical) OR (studies, historical cohort) OR (incidence studies) OR (incidence study) OR (studies, incidence) OR (study, incidence) OR (longitudinal study) OR (studies, longitudinal) OR (study, longitudinal) OR (tuskegee syphilis study) OR (syphilis studies, tuskegee) OR (syphilis study, tuskegee) OR (tuskegee syphilis studies) OR (jackson heart study) OR (heart studies, jackson) OR (heart study, jackson) OR (jackson heart studies) OR (studies, jackson heart) OR (california teachers study) OR (california teachers studies) OR (studies, california teachers) OR (study, california teachers) OR (teachers studies, california) OR (teachers study, california) OR (bogalusa heart study) OR (bogalusa heart studies) OR (heart studies, bogalusa) OR (heart study, bogalusa) OR (studies, bogalusa heart) OR (study, bogalusa heart) OR (framingham heart study) OR (framingham heart studies) OR (heart studies, framingham) OR (heart study, framingham) OR (longitudinal survey) OR (longitudinal surveys) OR (survey, longitudinal) OR (surveys, longitudinal))
3-	((“Cardiovascular Diseases”[Mesh]) OR (“Heart Diseases”[Mesh]) OR (“Cardiovascular Diseases”[Mesh]) OR (cardiovascular disease) OR (disease, cardiovascular) OR (diseases, cardiovascular) OR (disease, heart) OR (diseases, heart) OR (heart disease) OR (cardiac diseases) OR (cardiac disease) OR (disease, cardiac) OR (diseases, cardiac) OR (“Stroke”[Mesh]) OR (strokes) OR (cerebrovascular accident) OR (cerebrovascular accidents) OR (CVA) OR (cerebrovascular accident)) OR (cvas) OR (cerebrovascular accident) OR (cerebrovascular apoplexy) OR (apoplexy, cerebrovascular) OR (vascular accident, brain) OR (brain vascular accident) OR (brain vascular accidents) OR (vascular accidents, brain) OR (cerebrovascular stroke) OR (cerebrovascular strokes) OR (stroke, cerebrovascular) OR (strokes, cerebrovascular) OR (apoplexy) OR (cerebral stroke) OR (cerebral strokes) OR (stroke, cerebral) OR (strokes, cerebral) OR (stroke, acute) OR (acute stroke) OR (acute strokes) OR (strokes, acute) OR (cerebrovascular accident, acute) OR (acute cerebrovascular accident) OR (acute cerebrovascular accidents) OR (cerebrovascular accidents, acute) OR (“Mortality”[Mesh]) OR (mortalities) OR (case fatality rate) OR (case fatality rates) OR (rate, case fatality) OR (rates, case fatality) OR (mortality, excess) OR (excess mortalities) OR (mortalities, excess) OR (excess mortality) OR (decline, mortality) OR (declines, mortality) OR (mortality declines) OR (mortality decline) OR (mortality determinants) OR (determinant, mortality) OR (mortality determinant) OR (determinants, mortality) OR (mortality, differential) OR (differential mortalities) OR (mortalities, differential) OR (differential mortality) OR (age-specific death rate) OR (age-specific death rates) OR (death rate, age-specific) OR (death rates, age-specific) OR (rate, age-specific death) OR (rates, age-specific death) OR (age-specific death rate) OR (death rate) OR (death rates) OR (rate, death) OR (rates, death) OR (mortality rate) OR (mortality rates) OR (rate, mortality) OR (rates, mortality))
4-	((“Echocardiography”[Mesh]) OR (transthoracic echocardiography) OR (echocardiography, transthoracic) OR (echocardiography, cross-sectional) OR (echocardiography, cross sectional) OR (cross-sectional echocardiography) OR (cross sectional echocardiography) OR (echocardiography, m-mode) OR (echocardiography, m-mode) OR (m-mode echocardiography) OR (m-mode echocardiography) OR (echocardiography, contrast) OR (contrast echocardiography) OR (2d echocardiography) OR (echocardiography, two-dimensional) OR (echocardiography, two dimensional) OR (echocardiography, 2d) OR (echocardiography, 2-d) OR (echocardiography, 2 d) OR (two-dimensional echocardiography) OR (two dimensional echocardiography) OR (2-d echocardiography) OR (2 d echocardiography))
5-	1 AND 2 AND 3 AND 4

**Table S2:** The Newcastle-Ottawa Quality Assessment Scale for cohort studies.

	Selection				Comparability	Outcome				Score
Included studies	Representativeness of the exposed cohort	Selection of the non-exposed cohort	Ascertainment of exposure	Outcome not present at start of study	Comparability of cohorts	Assessment of outcome	Follow-up (yes or now)	Duration of follow-up (median)	Adequacy of follow-up	Number of stars
Lind et al. (9)	A*	A*	A*	A*	AB**	B*	A*	10	B*	9
Desai et al. (10)	A*	A*	A*	A*	AB**	A*	A*	10	B*	9
Lieb et al.(11)	AA*	A*	A*	A*	AB**	B*	A*	12	B*	8
Armstrong et al. (12)	A*	A*	A*	A*	AB**	B*	A*	20	D	8
Lai et al. (13)	A*	A*	A*	A*	AB**	A*	A*	14.4	D	8
Krumholz et al. (14)	A*	A*	A*	A*	AB**	A*	A*	8	D	8
Levy et al. (15)	A*	A*	A*	A*	AB**	A*	A*	4	A*	9
Nayor et al. (16)	A*	A*	A*	A*	A*	A*	A*	7.9†	B*	8
AlJaroudi et al. (17)	A*	A*	A*	A*	AB**	B*	A*	7.3	D	8
Desai et al. (18)	A*	A*	A*	A*	AB**	A*	A*	20	D	8
Kardys et al.(19)	A*	A*	A*	A*	AB**	A*	A*	3†	D	8
Bombelli et al. (20)	A*	A*	A*	A*	AB**	A*	A*	12.3	C	8
Armstrong et al. (21)	A*	A*	A*	A*	AB**	B*	A*	20	D	8
Kizer et al. (22)	A*	A*	A*	A*	AB**	A*	A*	7†	B*	9
Laukkanen et al. (23)	A*	A*	A*	A*	B*	A*	A*	13†	D	7
Tsang et al. (24)	A*	A*	A*	A*	AB**	B*	A*	3.8†	A*	9
Cuspidi et al. (25)	A*	A*	A*	A*	AB**	B*	A*	12.3	D	8
Lai et al. (26)	A*	A*	A*	A*	AB**	A*	A*	11.9	D	8
Gardin et al. (27)	A*	A*	A*	A*	AB**	A*	A*	9	D	8
Völzke et al. (28)	A*	A*	A*	A*	AB**	A*	A*	8.6	B*	9
Kizer et al. (29)	A*	A*	A*	A*	AB**	A*	A*	7†	D	8
Gardin et al. (30)	A*	A*	A*	A*	AB**	A*	A*	6.5†	D	8

Representativeness of the exposed cohort: A* means- truly representative.

Selection of the non-exposed cohort: A* means- drawn from the same community as the exposed cohort.

Ascertainment of exposure: A* means- secure record.

Outcome not present at start of study: A* means- yes.

Comparability of cohorts: AB** means: A*- the study controls for age, sex and marital status, B*- study controls for other factors.

Assessment of outcome A* means: independent blind assessment, B* means: record linkage.

Follow-up (yes or now): A* means- yes.

Adequacy of follow-up of cohorts: A* means- complete follow-up all subject accounted for, B* means- subjects lost to follow-up unlikely to introduce bias, C means- follow-up rate less than 80% and no  description of those lost, D means- no statement.

Good quality: 3 or 4 stars in selection domain AND 1 or 2 stars in comparability domain AND 2 or 3 stars in outcome/exposure domain.

† =Mean.

**Table S3:** Methods used to assess echocardiographic abnormalities, cardiovascular outcomes and risk found in the selected studies.

Study	Echocardiographic parameter	Outcomes	Risk
Lind et al. (9)	LVMI (height^2.7^) M-mode	MI, Stroke, HF, and Death (163)	Concentric LVH: HR=1.02 (1.003-1.03), *p*=0.019
Desai et al. (10)	LVMI (BSA) M-mode	CVD (CHD, HF, and MI) (542)	HR=1.26 (1.15-1.33) *p*<0.001
Lieb et al. (11)	LV mass M-mode	MI, HF, and CV death (140)	HR=1.59 (1.04–2.43)
Armstrong et al. (12)	LVMI (height^2.7^) LVMI (BSA)M-mode	HF, MI, Stroke, and death (118)	LVMI (height^(2.7)^: HR=1.18 (1.03-1.35) LVMI (BSA): HR=1.21 (1.05-1.39)
Lai et al. (13)	LV mass M-mode	CVD (MI and stroke) (205)	HR=2.01(1.11-3.63)
Krumholz et al. (14)	LVMI (height) M-mode	Total CV events (399)All-cause mortality (259)	M: HR=2.1 (1.5-3.1) W: HR=1.6 (1.0-2.6)
Levy et al. (15)	LV mass M-mode	Total CV events (208)	M: RR=1.49 (1.20-1.85) W: RR=1.57 (1.20-2.04)
Nayor et al. (16)	DD: (lateral e', E/A, E/e' age and sex-specific criteria) Pulsed wave Doppler and TDI	Total CV events (213)	DD moderate to severe HR=1.65 (1.14-2.38)
AlJaroudi et al. (17)	DD: Pulsed wave Doppler (transmitral influx, pulmonar venous flow) and TDI	All-cause mortality (71)	HR=2.03(1.07-3.84)
Desai et al. (18)	DD: Pulsed wave Doppler (E/A ratio)	All-cause mortality, MI, HF, and Stroke (149)	DD severe HR=4.3 (2.0-9.3), *p*=0.001 DD mild HR=1.6 (1.1-2.5), *p*=0.03
Kardys et al. (19)	DD: Pulsed wave Doppler (E, A**,** E/A, TD)	All-cause mortality (226)	E/A HR=1.49 (1.12–1.98)
Bombelli et al. (20)	LA diameter M-mode	Total CV events (198)	HR=2.1 (1-4.1), *p*=0.036
Armstrong et al. (21)	LA diameter and area (indexed to BSA and height) M-mode	Total CV events (226)	LAD: HR=1.34 (1.12-1.60) LAA: HR=1.43 (1.13-1.80)
Kizer et al. (22)	LA diameter M-mode	Total CV events (368)	HR=1.57 (1.17-2.10), *p*=0.002
Laukkanen et al. (23)	LA diameter (indexed to BSA and height) M-mode	CV death (54)	RR=1.5 (0.8- 4.1), *p*=0.15
Tsang et al. (24)	LA volume (indexed to BSA) Biplane area-length	Total CV events (333)	LAD: HR=1.29 (1.19-1.40), *p*<0.001
Cuspidi et al. (25)	Ao root diameter M-mode	Total CV events (137)	HR=2.62 (1.19–5.75), *p*=0.01
Lai et al. (26)	Ao root diameter (indexed to BSA) M-mode	CV events (185)All-cause mortality (335)	RR=0.76 (0.52-1.1), *p*=0.12RR=1.88 (1.04-3.40)
Gardin et al. (27)	Ao root diameter M-mode	All-cause mortality (581)	HR=M: 1.09 (0.83-1.42) HR=W: 1.30 (1.00-1.69)
Völzke et al. (28)	AV sclerosis and MAC M-mode and 2D	CV death=80, all-cause mortality (148)	AV sclerosis HR=1.87(1.12-3.11), *p*<0.017MAC HR=3.08(1.72-5.49), *p*<0.001
Kizer et al. (29)	AV sclerosis and MAC M-mode and 2D	Stroke=86	AV sclerosis RR=1.15 (0.45-2.49)MAC RR= 3.12 (1.77–5.25)
Gardin et al. (30)	MAC M-mode	CV events	MACCHD: HR=1.41 (1.04-1.93)CHF: HR=1.89 (1.29-2.79)

LVMI, left ventricular mass index; m-mode, unidimensional; MI, myocardial infarction; HF, heart failure; LVH=left ventricular hypertrophy; HR, hazard ratio; BSA, body surface area; CVD, cardiovascular disease; CHD, coronary heart disease; LV, left ventricle; CV, cardiovascular; RR, risk ratio; DD, diastolic dysfunction; TDI, tissue Doppler imaging; M, male; W, woman; LA, left atrial; LAD, left atrial diameter; LAA, left atrial area; Ao, aorta; AV, aortic valve; MAC, mitral annular calcification; CHF, congestive heart failure.
